# Ceruloplasmin in Parkinson's disease and the nonmotor symptoms

**DOI:** 10.1002/brb3.995

**Published:** 2018-05-07

**Authors:** Xuemei Zhao, Ziqiang Shao, Yu Zhang, Fang Liu, Zhibo Liu, Zunjing Liu

**Affiliations:** ^1^ Department of Radiology The First Hospital of Tsinghua University Beijing China; ^2^ Department of Neurology China‐Japan Friendship Hospital Beijing China; ^3^ The School of Medicine Tsinghua University Beijing China; ^4^ Department of Neurology The First Hospital of Tsinghua University Beijing China

**Keywords:** ceruloplasmin, motor symptoms, nonmotor symptoms, Parkinson's disease

## Abstract

**Objectives:**

To investigate the relationship between ceruloplasmin (CP) and Parkinson's disease (PD), and the correlation between CP level and the time difference between nonmotor symptoms and motor symptoms and the diagnosis were also mentioned.

**Materials and Methods:**

Sixty‐six patients diagnosed with PD for the first time were included in the study. They were divided into CP reduction group (31 cases) and CP normal group (35 cases) according to their CP level. The estimated time difference between nonmotor symptoms and motor symptoms and the diagnosis were recorded respectively. The magnetic sensitive nigra phase value was measured by susceptibility weighted imaging (SWI).

**Results:**

Ceruloplasmin level was middling correlated with age (*r* = .561, *p* < .001). There was strong negative correlation between CP level and UPDRS scores (*r* = −.727, *p* < .001). The CP level was significantly correlated with the magnetic sensitive nigra phase value (*r* = .891, *p* < .001). CP level showed moderate correlation with the time difference from nonmotor symptoms to motor symptoms (*r* = .559, *p* < .001), besides, the time difference between nonmotor symptoms and the diagnosis (*r* = .525, *p* < .001) and CP level was also moderately related.

**Conclusions:**

Ceruloplasmin interference in iron metabolism was closely related with PD development. And there were slight corrections between CP level and the time difference from nonmotor symptoms to motor symptoms or the diagnosis.

## 1 INTRODUCTION

Parkinson's disease (PD) is the second most common neurodegenerative disease in the world, second only to Alzheimer's disease (Toulouse & Sullivan, 2008). Clinical manifestations of PD include both motor and nonmotor symptoms. Muscle rigidity, resting tremor, slow movement, and postural disorders (Poewe et al., 2017) are the main motor symptoms and the nonmotor symptoms include disorder of sensation, sleep, emotion, cognition, and autonomic nerve and so on. Relja (2012) reported that nonmotor symptoms often tend to precede the onset of motor symptoms and accompany the patient's entire course of disease. The exact etiology of PD has not been elucidated, there may be a common impact of a variety of mechanisms. Genetic factors, environmental factors, abnormal protein expression, oxidative stress, abnormal iron metabolism, ubiquitin proteasome system, and autophagy are currently recognized as influencing factors of PD pathogenesis.

In recent years, excessive iron deposition was found in the substantia nigra of some Parkinson patients, which may be related to the pathogenesis and progression of PD (Jin et al., 2011). The abnormality of iron metabolism and related proteins, oxidative stress, and free radical generation induced by overdeposition of iron may be important reasons for the pathogenesis of PD (Jin et al., 2012). Hongge et al. (2009) reported that the incidence of PD was associated with the increase of local iron levels. Oakley et al. (2007) proved that the iron content of substantia nigra (SN) was abnormally deposited and the number of residual neurons was not related to iron content. It indicated that abnormal iron deposition was not secondary change after neuronal apoptosis, and it further confirmed that abnormal iron deposition in substantia nigra neurons was a specific change of PD (Ayton & Lei, 2014).

Ceruloplasmin (CP) was first isolated from pig serum in 1950s. The human CP gene which is located on chromosome 8, is a single polypeptide chain composed of 1,046 amino acids with molecular weight of about 132 kD, and it is the only copper oxidase in human body. CP is mainly synthesized in the liver, but large number of CP genes (Klomp & Gitlin, 1996) are expressed in human and rat astrocytes, especially perivascular astrocytes. In addition, CP plays a major role in copper transport due to its metal oxidation activity. It can promote the oxidation of Fe^2+^–Fe^3+^ and the synthesis of the transferrin, it also can catalyze the oxidation of adrenaline, dopamine, and other biological amines so as to reduce 6‐OHDA and other toxic metabolites (Waggoner et al., 1999). Therefore, the metabolism of iron in the brain is closely related to CP. Lower CP level in patients with movement disorders including PD was reported (Lirong et al., 2009). Further studies showed significant iron deposition in the substantia nigra of PD patients, and the iron content of other brain regions and the healthy control group revealed no significant difference. But the content of iron deposition in PD patients with hypoceruloplasminemia was significantly higher than patients with normal serum CP concentration and the control group and it was unrelated to the phenotype of motor symptoms (tremor‐dominant type, rigidity type, mixed type; Jin et al., 2012). Therefore, CP metabolism abnormalities may be a risk factor of PD and associated with iron deposition (Ayton et al., 2014).

This study aimed to investigate the relationship between CP and PD population in China, in addition, correlation between CP level and time difference from non‐motor symptoms to motor symptoms and the diagnosis were also mentioned.

## 2 MATERIAL AND METHODS

### 2.1 Subjects

From January 1, 2014 to December 31, 2016, 66 PD patients with first diagnosed PD were recruited in this study. They were divided into CP reduction group (31 cases) and CP normal group (35 cases) according to their CP level. All patients met the diagnostic criteria for PD of British PD society brain bank (Hughes, Daniel, Kilford, & Lees, 1992); PD was first confirmed, and no PD treatment was performed before this study. Additionally, we excluded patients with secondary PD or PD superposition syndrome or patients who had taken drugs for the treatment of PD before. Patients with family history or a history of blood transfusion or a plasma treatment had been performed before this study, or patients who had obvious internal organs (liver, kidney, heart, blood, etc.) dysfunction or patients with suspected or diagnosed tumor or acute and chronic inflammation were also excluded. Patients with metal material in the body or long‐term exposure of heavy metals or long engaged in chemical industry, metallurgical industry, patients with apparent abnormalities in intelligence, cognition, or spirit, and patient who cannot be followed were also excluded. This study was approved by the ethics committee of our hospital, all patients or their parents have signed the informed consent.

We asked the detail of the patient's history, the most likely first episode of nonmotor symptoms was determined, the estimated time difference from the first episode of nonmotor symptoms to the emergence of motor symptoms (referred to as pr‐exercise) and the estimated time difference between the first episode of nonmotor symptoms and the diagnosis of PD (Referred to as the pr‐diagnosis) was recorded. CP levels were determined, and CP ≥ 0.25 mg/ml was considered as normal, and CP < 0.25 mg/ml was regarded as a reduced level UPDRS score were evaluated in all patients as an assessment of PD. The phase values of the substantia nigra were measured by head susceptibility weighted imaging (SWI).

### 2.2 CP determination

At 7:30 a.m. in the morning, 3 ml of venous blood in the fasting state of all patients was extracted and sent to the laboratory, CP level was determined according to the turbidimetric method (BECKMAN COULTER IMMAGE800 biochemical analyzer).

### 2.3 Substantia nigra phase determination

Eight‐channel phased array coil was fixed to the head and neck, using GE 3.0T magnetic resonance imaging system. The patient's head was positioned in the midline between both shoulders. Cushions were used to help to maintain the head position.

Quadrature head coil was used, spin echo sequence (SE), axial and sagittal TlWI (TR635 ms, TE23.4 ms), axial T2WI (TR4600 ms, TE110 ms), and FLAIR sequence (TR9602 ms, TE117 ms) was included, the thickness was 5 mm, spacing was 1.5 mm. The axial MR scan had same level of positioning. SWI imaging parameters were as follows: SWI sequence: TR34.0 ms, TE20.0 ms, 41.67 Hz bandwidth, 448 * 448 matrix, 2.0 mm thickness, spacing was 0, vision (FOV) was 24 mm, flip angle was 15°.

The images were transmitted to the GEadw4.4 workstation, and then the post‐processing and data measurement of the images were carried out. According to SWI phase diagram, the substantia nigra phase values were measured by mouse in the axial position, all cases of the ROI of the region were blind measured by two radiologists at different times independently, three times repeated, and then the average values were obtained.

### 2.4 Statistical analysis

All data were analyzed by SPSS 22.0 (Chicago, IL, USA). After Kolmogorov–Smirnov test, the normal distribution data were analyzed by independent *T* test and Pearson correlation test. The non‐normal distribution data were analyzed by Mann–Whitney *U* test and Spearman's Rank Correlation Test, fisher exact probability method was used to count data. The normal distribution data was expressed as the mean ± standard deviation, and the data of the non‐normal distribution was represented by the median value (25% digits, 75% digits). *p* value < .05 was considered statistical significance.

## 3 RESULTS

The baseline characteristics and the constitution of the first onset of non‐motor symptoms of PD patients were showed in Table 1. Age between the CP reduction group and CP normal group was significantly different (*p* < .001).

**Table 1:** The baseline characteristics and the constitution of the first onset of nonmotor symptoms of patients of Parkinson's disease

Characteristics	CP normal group (*N* = 35)	CP reduction group (*N* = 31)
Age (years)	65.89 ± 5.38	56.35 ± 8.40
Gender male/female	18/17	22/9
Astriction	3 (8.6%)	1 (3.2%)
Sleep disorders	12 (34.3%)	5 (16.1%)
Pain	11 (31.4%)	9 (29%)
Olfactory dysfunction	2 (5.7%)	3 (9.7%)
Depression and anxiety	7 (20%)	13 (41.9%)

CP, ceruloplasmin.

There were significant difference between CP reduction group and CP normal group in CP level, magnetic sensitive nigra phase value, the time from non motor symptoms to motor symptoms (TNMS‐MS), the time difference between non‐motor symptoms and the diagnosis (TNMS‐D), UPDRS score respectively (all *p* < .001; Table 2).

**Table 2:** The outcomes and other results

	CP normal group (*N* = 35)	CP reduction group (*N* = 31)	*p*
CP level (mmol/ml)	0.69 ± 0.12	0.15 ± 0.04	<.001
MSNPV	−0.18 ± 0.02	−0.36 ± 0.17	<.001
TNMS‐MS (month)	17.91 ± 9.77	6.10 ± 3.13	<.001
TNMS‐D (month)	24.11 ± 12.19	9.06 ± 4.76	<.001
UPDRS score	14.74 ± 2.48	22.90 ± 5.27	<.001

CP, ceruloplasmin; MSNPV, magnetic sensitive nigra phase value; TNMS‐D, the time between nonmotor symptoms and the diagnosis (month); TNMS‐MS, the time from nonmotor symptoms to motor symptoms (month); UPDRS score, score of unified Parkinson diease rating scale.

Correlation analysis results showed that CP levels were moderately correlated with age (*r* = .561, *p* < .001). The CP level and UPDRS scores (*r* = −.727, *p* < .001) were negatively correlated. The CP level was significantly correlated with the magnetic sensitive nigra phase value (*r* = .891, *p* < .001).

There was a moderate correlation between CP levels and the time difference from non‐motor symptoms to motor symptoms (*r* = .559, *p* < .001) and the time difference between non‐motor symptoms and the diagnosis (*r* = .525, *p* < .001) and CP level was also moderately related.

## 4 DISCUSSION

Parkinson's disease is a common disease of the central nervous system in elderly population, characterized by myotonia, tremor, bradykinesia, postural instability, gait difficulties and the main pathological changes of PD was the death of dopaminergic neuron in the substantia nigra (Xuan, Yongli, Huijuan, & Yuqiao, 2011). Research showed increased brain iron content in patients with Huntington's disease, PD, Alzheimer's disease, multiple sclerosis, cerebral hemorrhage, cerebral infarction, chronic anemia, thalassemia, hemochromatosis, and other diseases (Bartzokis & Tishler, 2000). Age is a recognized risk factor of PD (Collier et al., 2007), this was confirmed in this study.

In the past, motor symptoms of Parkinson were focused on, while it has been rarely mentioned, but these non‐motor symptoms can seriously affect the life quality of patients with PD (Gardner et al., 2017; Mu et al., 2017; Muzerengi, Contrafatto, & Chaudhuri, 2007). All PD patients basically had different degrees of sleep disorders, most of them occurred at the early stages (Chaudhuri, 2003). Related reports in patients with PD showed that the occurrence of a variety of sleep disorders ranged from 25% to 98% (Schrag, Ben‐Shlomo, & Quinn, 2002), which is consistent with 17 (25.8%) patients with sleep disorders in this study. It (Wood, Bilclough, Bowron, & Walker, 2002) has been reported that depression was occurred in 10%–45% PD patients, this is consistent with the results that 20 (30.3%) patients with depression and anxiety in this study. Olfactory function became apparent before any motors symptoms, which may be caused by local metal imbalance (Gardner et al., 2017). But may be due to the less number of cases in this study, the percentage of olfactory dysfunction in the study was 5 (7.6%).

Susceptibility‐weighted imaging is a new MRI technique that use signal differences in magnetic susceptibility of various tissues to make signal contrast (Wang, Liu, & Wang, 2010), and is more sensitive to iron. It uses the difference imaging of magnetic susceptibility due to different iron content among tissues (Tao, Zhou, & Ge, 2009). SWI image has a good resolution to the small anatomical structure in the brain, which can clearly distinguish gray and white matter, increase the contrast between the two, and thus it can well show the nuclei of Parkinson's patients (Haller et al., 2013; Meijer et al., 2016) accompanied by abnormal iron deposition (Zhang et al., 2010). These nuclei have lower signal, better uniformity, and sharp boundary in SWI. In addition, it was found that (Zhang & Zhang, 2009) the iron content in the brain has curve linear relationship with SWI phase diagram. The calibration phase map on the phase value of the measurement can reflect the iron content quantitatively, which can help us to understand the progress of the disease and predict the effect of the treatment. And in this study, CP level was significantly and strongly correlated with the magnetic sensitive nigra phase value.

Ceruloplasmin is the only copper oxidase in humans and vertebrates, which can oxidize Fe^2+^–Fe^3+^ and promote the combination of iron and Tf (McAuley et al., 2010). Recent studies have found that CP is also expressed in mammalian CNS and can be synthesized in the brain, and presumably involved in normal brain iron metabolism (Ayton et al., 2014; Pretorius, Bester, & Kell, 2016). decreased serum CP concentration in PD patients was confirmed (Tórsdóttir, Kristinsson, Sveinbjörnsdóttir, Snaedal, & Jóhannesson, 1999). Wang (2005) observed a 64% decrease in the expression of CP in the damaged side of SN compared with the normal control, in PD‐model rats prepared by 6‐OHDA. High iron content in neuronal degenerative diseases such as PD and decrease of CP in cerebrospinal fluid and plasma may be due to the increase of nontransferrin binding iron (NTBI) and the reduction of released iron (Wang, 2005). Under pathological condition, the activity of CP oxidase decreased, which made Fe^2+^ oxidized to Fe^3+^, and Fe^3+^ and Fe‐Tf decreased, while NTBI and free Fe^2+^ increased, hence brain cells increased NTBI uptake. In addition, in spite of the increase in intracellular Fe^2+^, Fe^2+^ cannot be released because of the decrease of iron concentration due to the increase of extracellular Fe^2+^. These factors contribute to the accumulation of iron and lead to oxidative stress, which eventually leads to neuronal death (Wang, 2005; Tórsdóttir et al., 1999).

In conclusion, the possibility of first episode of nonmotor symptoms in PD patients was very high, sleep disorders, pain, depression, and anxiety needed to be paid attention to. CP played an important role in the iron metabolism in the body, which was closely related to the development of PD. The limitation of this study is the number of cases was less, further study with big sample size is encouraging.

## CONFLICT OF INTEREST

None declared.
